# Vitamin D and Bone Disease

**DOI:** 10.1155/2013/396541

**Published:** 2012-12-27

**Authors:** S. Christodoulou, T. Goula, A. Ververidis, G. Drosos

**Affiliations:** Department of Orthopaedic Surgery, School of Medicine, Democritus University of Thrace, University General Hospital of Alexandroupolis, Dragana, 68100 Alexandroupolis, Greece

## Abstract

Vitamin D is important for normal development and maintenance of the skeleton. Hypovitaminosis D adversely affects calcium metabolism, osteoblastic activity, matrix ossification, bone remodeling and bone density. It is well known that Vit. D deficiency in the developing skeleton is related to rickets, while in adults is related to osteomalacia. The causes of rickets include conditions that lead to hypocalcemia and/or hypophosphatemia, either isolated or secondary to vitamin D deficiency. In osteomalacia, Vit. D deficiency leads to impairment of the mineralisation phase of bone remodeling and thus an increasing amount of the skeleton being replaced by unmineralized osteoid. The relationship between Vit. D and bone mineral density and osteoporosis are still controversial while new evidence suggests that Vit. D may play a role in other bone conditions such as osteoarthritis and stress fractures. In order to maintain a “good bone health” guidelines concerning the recommended dietary intakes should be followed and screening for Vit. D deficiency in individuals at risk for deficiency is required, followed by the appropriate action.

## 1. Introduction

Vitamin Dis important for normal development and maintenance of the skeleton. It is well known that Vit. D deficiency is related to rickets and osteomalacia. The relationship between Vit. D and bone mineral density and osteoporosis are still controversial while new evidence suggest that Vit. D may play a role in other bone conditions such as osteoartrhitis and stress fractures.

After a short introduction of Vit. D sources, metabolism, and serum levels, this paper reports the recent evidence concerning the relationship between Vit. D and condition affecting the human skeleton. 

### 1.1. Vitamin D Sources and Metabolism

Vitamin D is available either as ergocalciferol (vitamin D2) or as cholecalciferol (vitamin D3). Ergocalciferol, which is derived from plants, is converted by the liver to 25-hydroxyvitamin D2 (25(OH)D2), then by the kidneys to 1,25-dihydroxyvitamin D2 (1,25(OH)2 D2) [1]. 

Similarly, cholecalciferol, from animal sources, is converted to 25(OH)D3 then 1,25(OH)2 D3. Cholecalciferol is abundant in a few food sources (e.g., fish liver) and is often used as a dietary supplement, either alone or with calcium [1]. 

Ultraviolet B (UV-B) radiation (290–315 nm) converts 7-dehydrocholesterol in the deep epidermal layers to the provitamin cholecalciferol. 

### 1.2. Vitamin D Serum Levels

Measurement of the active form 1,25 (OH)2D is not useful in clinical practice. The serum 25(OH)D level reflects the vitamin D stores in the body. Studies have shown considerable interindividual variability in 25(OH)D levels related to differences in sunshine exposure, clothing style, skin pigmentation, skin thickness, age, and weight (with lower levels in heavier individuals due to storage of vitamin D in fat). The 25(OH)D level dips in winter and increases in summer [2].

The normal 25(OH)D values remain ill-defined and no consensus exists concerning the minimum serum concentration of 25-hydroxyvitamin D necessary to guarantee optimal health.

Normal serum 25(OH)D values have been defined as >20 ng/mL (50 nmol/L). Deficiency is defined if serum values are <20 ng/mL (50 nmol/L) and severe deficiency if they are <21–29 ng/mL (52.5–72.5 nmol/L). Serum values of 25(OH)D >200 ng/mL (500 nmol/L) are considered as toxic [3].

We recommend using the serum circulating 25(OH)D level, measured by a reliable assay, to evaluate vitamin D status in patients who are at risk for vitamin D deficiency. Vitamin D deficiency is defined as a 25(OH)D below 20 ng/mL (50 nmol/liter) and vitamin D insufficiency as a 25(OH)D of 21–29 ng/mL (52.5–72.5 nmol/liter). We recommend against using the serum 1,25(OH)2D assay for this purpose and are in favor of using it only in monitoring certain conditions, as acquired and inherited disorders of vitamin D and phosphate metabolism.

### 1.3. Vitamin D and Calcium Intake

Vit. D, PTH, and Ca levels are related. Dietary calcium intake influences the PTH level, and, in turn, variations in PTH levels can influence the turnover rate of vitamin D metabolites [4].

Low calcium intake is associated with elevations in PTH and 1,25(OH)2 D levels and with a decrease in 25(OH)D half-life. Thus, calcium deficiency may worsen vitamin D deficiency, whereas a high calcium intake may exert a vitamin D sparing effect [5].

### 1.4. Vitamin D and Intestinal Absorption of Calcium

Vitamin D has long been recognized as important for calcium absorption, but the quantitative relationships between intake, vitamin D status, and absorption are less well documented.

Whether 25(OH)D directly influences absorption is unclear, variations in 25(OH)D levels are not associated with significant variations in 1,25(OH)2 D levels, and in osteomalacia, calcium absorption may be profoundly diminished despite normal or elevated levels of 1,25(OH)2 D [6]. It has been suggested that calcium malabsorption may occur only when the 25(OH)D level is insufficient to maintain the 1,25(OH)2D level despite secondary hyperparathyroidism [7].

Uncertainties remain, however, about the relation between the 25(OH)D level and calcium absorption.

 In healthy men, calcium absorption showed very little difference when the 25(OH)D level decreased from 122 nmol/L after a summer of outdoor activity (the equivalent of 2800 IU vitamin D per day) to 74 nmol/L in late winter [8].

In one study, calcium absorption was measured from the area under the curve of the serum calcium increase induced by an oral calcium load in the spring, with and without pretreatment with 25(OH)D [9]. The serum 25(OH)D level was 86.5 ± 25 nmol/L with pretreatment and 50.2 ± 15 nmol/L without pretreatment; both ranges were considered normal. All participants received 500 mg of calcium per day orally. Calcium absorption efficiency was 65% greater with than without pretreatment and the authors concluded that the lower 25(OH)D values (50.2 ± 15 nmol/L) were associated with suboptimal calcium absorption [9].

 Calcium absorption efficiency may improve with rising 25(OH)D concentrations up to 80 nmol/L and level off subsequently, according to clinical studies that assessed the absorption efficiency as a function of vitamin D status [9, 10]. A clinically important improvement in absorption efficiency as serum 25(OH)D dose from 50 to 66 nmol/L [9] and to 83 nmol/L [8]. Furthermore, the rate of rise per unit change in serum 25(OH)D was identical for both studies [9, 10]. In a third study, only a very small difference in absorption efficiency was noted in individuals studied at 25(OH)D levels of 120 and 78 nmol/L. [8]. Taken together, these studies may suggest that absorption efficiency rises up to serum 25(OH)D levels of 80 nmol/L, with a plateau occurring at higher levels.

## 2. Rickets and Vitamin D

Normal bone growth and mineralization depends on the availability of adequate calcium and phosphate. Deficient mineralization at the growth plate can result in rickets. Rickets usually occur as long as the growth plates are open as in children [11].

The mineralization defects can be classified as calcipenic (hypocalcemic) rickets caused by calcium deficiency and phosphopenic (hypophosphatemic) rickets caused by phosphate deficiency [12].

Vitamin D is a prohormone that is essential for normal absorption of calcium from the gut, and deficiency of vitamin D is usually more common than either isolated calcium or phosphorus deficiency and is the commonest cause of rickets. The causes of rickets include conditions that lead to hypocalcemia and/or hypophosphatemia, either isolated or secondary to vitamin D deficiency [13].

### 2.1. Calcipenic Rickets

Calcipenic (hypocalcemic) rickets is characterized by deficiency of calcium. Rickets can occur despite adequate vitamin D levels if the calcium intake is very low. This problem generally does not occur unless calcium intake is very low because vitamin D increases intestinal calcium absorption. Most children with calcium deficiency rickets have normal serum 25-hydroxy vitamin D [25(OH)D] and high serum 1,25-dihydroxy vitamin D [1,25(OH)_2_] concentrations, indicating adequate intake of vitamin D. These children may have an increased vitamin D requirement when measured by their response to vitamin D replacement. Thus, vitamin D requirements may be higher than those expected in children who are calcium deficient [14].

In addition to this, low dietary calcium intake even without coexisting vitamin D deficiency increases serum 1,25(OH)_2_D concentrations, which in turn decrease the half-life of 25(OH)D, probably by increasing the catabolism of 25(OH)D [15].

## 3. Osteomalacia and Vitamin D

Osteomalacia arises from a disorder in the physiological process of bone turnover where the mineralisation phase of bone remodeling is impaired. Whenever vitamin D deficiency is implicated in the etiology of osteomalacia there is usually evidence of secondary hyperparathyroidism [16].

An insufficiency of either calcium or vitamin D can lead to prolonged secondary hyperparathyroidism and as a result lead to cortical porosity and thinning of cortical bone arising from a net loss of cortical bone on the endosteal surface. This loss of cortical bone is irreversible, whereas trabecular bone mass is relatively well maintained in situations of parathyroid hormone excess [16].

An absolute deficiency of vitamin D, however, leads to an increasing amount of the skeleton being replaced by unmineralized osteoid. As a result weight-bearing bones begin to bend, and although this is imperceptible, it is painful because the periosteum is stretched and the patient experiences vague pains in the limbs. The serum calcium may fall because calcium absorption is impaired due to low or absent 1,25(OH)2D levels, and because large areas of the bone surfaces are covered by osteoid, calcium release from bone is impaired [17].

There are three phases that comprise hypovitaminosis D osteopathy (HVO). The first phase, HVOi, results from secondary hyperparathyroidism with an increase in osteoid accumulation due to increased bone turnover. This stage defines as preosteomalacia. Osteomalacia is defined as either HVOii or HVOiii. In HVOii there is a delay in mineralization of osteoid such that only the earliest formed matrix becomes mineralized, and in HVOiii none of the matrix formed becomes mineralized. These three histological variants probably represent the development of vitamin D deficiency, leading to a lack of vitamin D, to maintain serum and tissue levels of 1,25(OH)2D [18].

Changes in bone histology must take many weeks or months to develop; changes to the circulating serum levels of vitamin D metabolites can occur within hours or days of exposure to solar irradiation or an oral supply of vitamin D. These factors can result in normal or raised serum levels of 1,25(OH)2D [19].

## 4. Vitamin D, Bone Mineral Density, and ****Osteoporosis

Adequate levels of vitamin D have an important effect. Hypovitaminosis D adversely affects calcium metabolism, osteoblastic activity, matrix ossification, bone remodeling, and bone density [20]. Low 25-hydroxyvitamin D (25OHD) associated with secondary hyperparathyroidism and increased bone turnover. Vitamin D deficiency can be an important risk factor for osteoporosis [21].

Bone mineral density (BMD), which measures the quantity of the calcified bone, at present is the gold standard technique for the diagnosis of osteopenia and osteoporosis. The association between 25OHD and BMD is still debatable. 

Differences across studies in vitamin D dosages and administration modalities complicate the interpretation of data on the relation between vitamin D status and BMD. Some studies suggest that a low serum 25OHD level is associated with low BMD [22, 23] and in postmenopausal women with osteoporosis, 25(OH)D levels correlated positively with various BMD parameters at a threshold of 50 nmol. 

However, no association between vitamin D status and BMD has been found in other studies [24–26] and vitamin D supplementation alone failed to effectively prevent bone loss in postmenopausal women, patients with osteoporosis, or glucocorticoid users [27]. Other studies found that vitamin D and calcium supplementation was associated with small reductions in the bone loss rate, of 0.54% at the hip and 1.19% at the lumbar spine [28].

It seems that the association of vitamin D status and bone mineral density is depending upon the study population and study design.

### 4.1. Response to Treatment

Inadequate response to bisphosphonate (BP) treatment is frequent in postmenopausal women with osteoporosis. Several factors have been implicated such as low adherence to treatment, low vitamin D intake, severity of the disease, older age, the type of antiresorptive agent used, differences in bioavailability, low dietary calcium intake, and the presence of underlying causes of secondary osteoporosis [29–31].

According to recently presented preliminary research findings, a better response to bisphosphonate (BP) treatment is expected with 25(OH)D serum values over 30 ng/mL [32–34].

In a Spanish study, Peris et al. investigated that 25(OH)D serum levels influence adequate response to bisphosphonates (BP) treatment in postmenopausal osteoporosis. Patients classified as inadequate responders to BP treatment, by BMD loss or the development of fractures, presented significantly lower levels of 25(OH)D and increased urinary NTx values, thereby suggesting an increase in bone turnover and, consequently, in bone loss in these patients. In addition, patients with 25(OH)D serum levels lower than 30 ng/mL also had a significantly lower increase in bone mass, further confirming the importance of maintaining the serum concentrations of 25(OH)D over this reference value, especially when inadequate response to BP is observed [34].

Inadequate response to BP treatment has also been observed despite vitamin D supplementation. For every 100 IU of supplementation, an elevation of 1 ng/mL of serum 25(OH)D is expected [35, 36]. However, factors such as baseline 25(OH)D serum concentrations, obesity, age, or associated clinical conditions, among others, may influence this response. All of this may probably explain the continued uncertainty as to the optimal dose regimens required in the general population, with vitamin D recommendations for postmenopausal women ranging from 600 to 1000 IU, the highest doses being especially indicated in overweight and more severely vitamin D-deficient individuals [37, 38].

Therefore, the requirements of vitamin D per day to achieve a serum level of 30 ng/mL may differ depending not only on the baseline serum levels of 25(OH)D but also on the characteristics of each individual. Indeed, recent data have indicated the need for larger doses of vitamin D in some individuals to suppress the presence of secondary hyperparathyroidism [39].

## 5. Vitamin D and Stress Fractures

Stress fractures are common in recruits and young athletes. Several predisposing factors have been suggested, but the precise pathogenetic role remains unclear. Changes in bone metabolism related to nutritional and hormonal changes possibly play an important role in the pathogenesis of stress fractures by changing the bone composition and probably by influencing the bone turnover. 

Dietary Vit. D deficiency, and low levels of Vit. D, as well as other parameters related to low vit. D levels such as elevated serum PTH concentration and decreased BMD, have been implicated [40–44]. 

Another study conducted among military personnel, furthermore, suggested that polymorphisms of the vitamin D receptor gene may be associated with increased risk of stress fractures [45].

A lower level of serum 25(OH)D concentration may be a predisposing factor for bone stress fractures [40–42], but controversy exists as far as the cut-off level is of concern.

A recent study found that the development of stress fractures during basic training was associated with dietary deficiency of mainly vitamin D and calcium before induction and during basic training [44].

## 6. Vitamin D and Osteoarthritis

Osteoarthritis (OA) is a chronic degenerative disorder characterized by cartilage loss. OA is considered today as a disease of the cartilage, synovial membrane, and the subchondral bone [46]. The pathogenesis of the disease is not clear, but recent evidence supports the theory that an imbalance of the subchondral bone remodeling may initiate the degenerative process [47].

Vit. D is related to bone turnover [48]. Low serum 25-OHD increases osteoblastic activity and bone turnover [48]. Therefore it has been suggested that** i**nsufficient levels of serum 25-hydroxyvitamin D may be related to changes in subchondral bone. These changes play an essential role in the onset and progression of cartilage lesions [48].

A significant association between serum 25-OHD deficiency and knee OA has been found [49, 50]. It has been suggested that serum 25-OHD shall be measured in any patients with symptoms suggestive of knee OA particularly at the initial stage of disease [49]. Although the level of 25(OH)D seems not to be related to the severity of the knee X-ray grading or to the functional assessment [50].

However, the findings of another study indicate thatvitamin Dstatus is unrelated to the risk of joint space or cartilage loss in knee OA [51].

It is still unclear whether there is a relationship between Vit. D status, BMD, and OA [52–54]. The general belief that OA “protects” from osteoporosis may not be true [52]. It has been found that a significant proportion of female hip OA patients (74%) had a low BMD (were osteopenic or osteoporotic) with signs of increased bone turnover [52]. The prevalence of Vit. D insufficiency in these patients was also relatively high [52].

A significant positive association between serum 25(OH)D and BMD in individuals with primary knee OA, independent of sex, age, BMI, knee pain, physical activity, and disease severity has been found [53].

A low dietaryvitamin Dintake increases the risk of progression of knee radiographic OA according to the results of another study [54]. Furthermore it was supported that improving thevitamin Dstatus in the elderly could protect against the development and worsening of knee OA, especially in those with low BMD [54].

## 7. In Conclusion

In order to maintain a “good bone health” guidelines concerning the recommended dietary intakes should be followed [55]. Although many controversies still exist, normal serum Vit. D levels are recommended [55]. 

A screening for Vit. D deficiency in individuals at risk for deficiency is required and the appropriate treatment is recommended keeping in mind that more vitamin D is not necessarily better [55].

The beneficial role of Vit. D in prevention of conditions such as osteoarthritis and stress fractures remains to be seen.
